# Implementing peer support work in mental health care in Germany: The methodological framework of the collaborative, participatory, mixed‐methods study (ImpPeer‐Psy5)

**DOI:** 10.1111/hex.13938

**Published:** 2023-12-19

**Authors:** Sebastian von Peter, Ute Maria Kraemer, Lauren Cubellis, Georgia Fehler, Guillermo Ruiz‐Pérez, Daniela Schmidt, Jenny Ziegenhagen, Madeleine Kuesel, Susanne Ackers, Candelaria Mahlke, Lena Nugent, Imke Heuer

**Affiliations:** ^1^ Brandenburg Medical School, Centre of Mental HealtPsychiatry and Psychotherapy Immanuel Klinik Rüdersdorf Neuruppin Germany; ^2^ EX‐IN Germany e.V. Karlsbad Germany; ^3^ Department of Psychiatry University Medical Center Hamburg‐Eppendorf Hamburg Germany

**Keywords:** collaborative, mental health care, mixed method, peer support work, participatory research

## Abstract

**Background:**

Starting in the 1990s in the United States, individuals with lived experience of mental health crises and recovery have been employed as peer support workers (PSWs) internationally. However, the implementation of PSW in clinical contexts remains challenging.

**Methods:**

This manuscript presents and discusses the methodological framework of the ImpPeer‐Psy5 study on the PSW implementation in the German mental healthcare sector. This study used a mixed‐methods and collaborative research approach, as well as participatory research strategies. After describing the study design, populations, teamwork and assessments, the epistemic challenges of its methodological framework will be critically discussed and how it has iteratively shaped the object of study.

**Discussion and Practical Implications:**

The healthcare, policy and funding context of PSW implementation as well as the study's methodological framework have differently influenced the ways in which the implementation of PSW has been conceived in this study. The choice of a collaborative or participatory methodological framework is advised to better align research questions and procedures to the specific needs and challenges of PSWs and other stakeholders concerned with PSW implementation.

**Patient and Public Contribution:**

The research team of the ImpPeer‐Psy5 study was collaboratively staffed by a portion of researchers who also identify as users or survivors of psychiatric services. A nonprofit organization for the training of PSWs served as a practice partner throughout the research process. Different participatory formats involve a significant number of diverse stakeholders relevant to PSW implementation.

## 1 BACKGROUND

For more than 30 years, individuals with lived experience of mental health crises and recovery have been involved in the provision of mental health care as peer support workers (PSWs). The implementation of PSW spans the United States of America, the United Kingdom, Australia, Canada, various European countries and, in recent years, several Asian and African countries. This expansion is supported by research evidence from various healthcare contexts on PSW effectiveness: for instance, a recent review by White et al. demonstrated that one‐to‐one PSW significantly improves psychosocial outcomes.^1^ The review by Smit et al. showed that PSW had consistent positive effects on both clinical and personal recovery.^2^ In relation to the German healthcare context, a randomized controlled trial demonstrated that support from PSWs significantly enhanced the self‐efficacy of service users.^3^ This fast‐growing evidence base justifies the inclusion of PSW in both national and international guidelines.^1^, ^4^, ^5^, ^6^ At the same time, numerous publications discuss the challenges of implementing PSW in clinical and other care contexts and how to evaluate these processes.^7^, ^8^, ^9^, ^10^, ^11^, ^12^, ^13^, ^14^, ^15^, ^16^, ^17^, ^18^, ^19^ Against this context, this manuscript presents and critically evaluates the methodological framework and epistemic challenges of a collaborative, participatory, mixed‐methods study on the implementation of PSW in the German mental healthcare sector.

Peer support in the field of mental health was originally developed across diverse strands of the consumer/survivor/(ex‐)patient/peer (=c/s/x‐) movement and was consequently grounded in self‐help principles such as mutuality, dignity, empowerment, reclaiming self‐determination etc.^20^ The implementation of PSW in clinical contexts occurred considerably later, bringing with it structured training programmes, formalized certifications and various policy concerns. This integration of peer support into mental health care has led to critical debates within the c/s/x‐movement, which have criticized the assimilation, commodification and cooptation of the original values and principles of peer support.^21^, ^22^ In this article, the expression ‘PSW’ has been chosen as a term to designate more formalized forms of peer support that are being integrated into healthcare services.

From the outset, the gradual integration of PSW into the healthcare system has internationally been accompanied by research on the challenges posed by employing this workforce.^11^, ^12^, ^13^, ^14^, ^15^, ^16^, ^17^, ^18^, ^19^ This research has revealed important insights in relation to PSW implementation, such as confusion regarding the definition of roles and tasks of PSWs.^23^, ^24^ Other results concern topics such as trainings for PSWs and the staff working alongside them, questions of how peer‐delivered services can be more clearly defined, discussions around peer‐specific supervisions, opportunities for professionalization and career development for this workforce.^25^, ^26^

This and other implementation studies have used a wide range of methodological frameworks, meaning sets of procedures, methods and tools that systematically guide and structure research processes, defining how to collect and analyse data, or draw conclusions from them.^27^, ^28^, ^29^ Some studies have explored and synthesized the salient factors of PSW implementation by qualitatively analysing the perceptions of various stakeholders.^13^, ^30^ Others have studied this topic using role adaption models.^31^, ^32^ More recently, multilevel frameworks have been developed, investigating PSW implementation in relation to wider political, economic and regulatory contexts.^17^, ^18^, ^33^ For instance, the systematic review and narrative synthesis by Ibrahim et al.^34^ identified eight facilitating factors and barriers to the implementation of PSW (organizational culture, PSW trainings, role definitions, staff's willingness and ability to work with PSWs, resource availability, financial arrangements, support for PSWs well‐being and their access to peer networks) and mapped these determinants onto the domains of the Consolidated Framework for Implementation Research (CIFR).

Methodological frameworks usually bring certain aspects of a research topic into the foreground, while relegating others to the background.^27^, ^35^ Thus, they should be chosen carefully, not only in the field of implementation research, but also concerning evaluative or outcome studies.^36^ In relation to PSW implementation, such a careful choice of the methodological framework and its critical problematization have been strongly recommended, as research on this topic risks assimilating or commodifying this workforce rather than supporting the preservation of its distinct values and modes of operation.^37^, ^38^ In this context, we highlight the need for the systematic participation of all stakeholders involved in processes of PSW implementation to better align the research with their needs and experiences, which, to our knowledge, has so far been implemented by few other studies, such as the ones by Jones et al and Byrne et al.^39^, ^40^

### 1.1 Aim and questions

Against this background, this manuscript details and critically discusses the methodological framework of our collaborative, mixed‐methods study ImpPeer‐Psy5, which explores the implementation of PSW in the German healthcare context. As PSW is adaptive to local contexts, the first section contains background information on the German healthcare system. Subsequently, the methodological framework used in our study will be described in some detail, followed by a discussion of the ways in which this framework has iteratively shaped the object of our study—the implementation of PSW in the German mental healthcare sector. Thus, this manuscript follows the question in how far the methodological choices of our study have explored and by thereby foregrounded certain ways of understanding the topic of PSW implementation. In that way, it follows the assumption that research is a social procedure that actively shapes the reality which is being studied.^37^ Thus, in the discussion, this question will be explored in relation to critical debates on the integration of PSW in mental healthcare systems internationally.

### 1.2 The study context

In 2005, in Germany and five other European countries, EU‐funding supported the basic development of the “Experienced Involvement” (EX‐IN) PSW training courses, which train individuals with lived experience of mental health diagnoses as PSWs. The programme was considered successful, and thus, was implemented throughout Germany. Currently, there are 29 EX‐IN training sites nationwide. The graduates of these trainings are certified as PSWs, with their qualifications being recognized by a wide range of mental health service providers in Germany. Further, there are various alternatives to the EX‐IN training, such as peer counselling courses provided by the German Network for Independent Living, which focuses on persons with disabilities (Peer Counsellor ISL),^41^, ^42^ as well as other programmes that are smaller in scope, such as UPSIDES or DBT Peer Coach.^42^, ^43^ At the same time, EX‐IN remains the dominant training paradigm, with the consequence that our study, despite efforts to recruit PSWs with varied training backgrounds, was largely dominated by EX‐IN graduates due to their greater numbers. However, several graduates of other programmes were also included.

In the German context, there are considerable regional and institutional differences in the implementation of PSW, which reflect the diversity of local, regional, as well as federal regulations and conditions. Public mental health care in Germany is mainly provided through psychiatric hospitals, of which there are roughly 600 throughout the country. These hospitals focus primarily on the provision of inpatient services, followed by a considerably smaller proportion of day‐clinics, outpatient services and a very small section of home treatment services. These services, and the services provided by licensed psychiatrists and psychologists/psychotherapists, are regulated by the Fifth German Social Code Book (SGB V) and funded by approximately 90 statutory health insurance companies.^44^ Around 70% of all expenditure spent on people with mental health diagnoses accounts for this sector,^45^, ^46^ which, in the remainder of this article, is labelled ‘mental healthcare sector’, also to distinguish it from the ‘psychosocial sector’, which includes a broad spectrum of complementary, vocational, residential and psychosocial services^47^ regulated by different Social Code Books (mainly SGB IX and XII) and funded by various bodies such as pension funds or individual social benefits. While there are no definite numbers, it is estimated that the majority of the approximately 2500 PSWs in Germany are employed in the latter (psychosocial) field. Yet, due to the aim of this study and its funding body (see below), it specifically evaluated the implementation of PSW in the mental healthcare sector, mainly focusing on PSWs employed in psychiatric hospital departments.

This focus was also justified by recent developments: in 2016, a new law was passed (the PPP‐RL law), aiming to restructure the planning of human resources and the remuneration system of the German mental health hospital sector. In this context, it was decided to include PSW as a new professional group. Thus, core tasks had to be defined and added to staffing guidelines. These developments have yielded opportunities for PSWs, as well as challenges, changing the conditions under which PSW is integrated in the service provision as well as the institutional and individual variables on the performance of their work tasks. Above all, the need for hospitals to develop strategies to implement PSW and integrate this new group of care workers in the mental healthcare sector has become a major concern, which provides the larger background for the implementation of the ImpPeer‐Psy 5 study.

## 2 THE IMPPEER‐PSY 5 STUDY

### 2.1 Design

#### 2.1.1 Study aims

The ImpPeer‐Psy5 study aimed to provide a situational analysis of the current state of barriers to, and requirements for the implementation of PSW in the German healthcare sector. Specifically, it proposed to answer the following research questions: (1) What training, working or implementation models exist for PSWs in the German mental healthcare system? (2) What needs, expectations, hopes and fears for PSW are articulated by the stakeholders involved and how do these perspectives affect the implementation of PSW? (3) Which dimensions of international PSW might be useful in Germany, and how can they be adapted to the German healthcare context to meet local needs and conditions?

#### 2.1.2 Mixed‐methods approach

Given the complex and multidimensional nature of the research topic, our study applied a mixed‐methods approach, aiming to align and synthesize various procedures and results from different study parts, research methods and strategies, as well as diverse experiences and perspectives. A mixed‐methods approach was used to enable the triangulation and contextualization of data and results, thereby increasing the credibility and generalizability of findings.^48^

#### 2.1.3 Study parts

**Figure 1:**
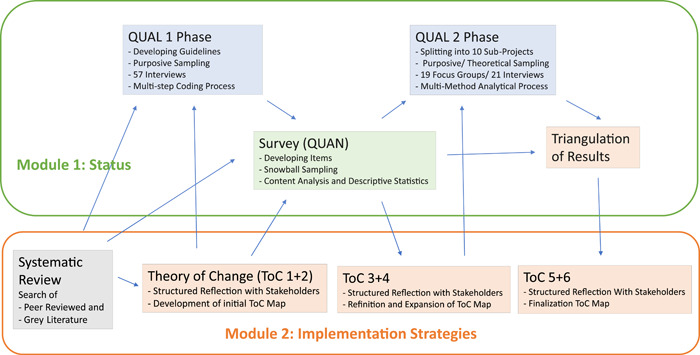
Interconnected work streams, using different data collection methods with the aim to integrate the results. For more details regarding the methodological design of all study phases, see sections below.

In the context of our study, methods were anchored in different disciplinary fields, and joined by the researchers' awareness that this methodological combination would strongly shape the knowledge produced—an assumption that is also at the heart of this article's discussion. Overall, the study used a QUAL–QUAN–QUAL design (see Figure 1) to contrast and balance diverging approaches and perspectives, as well as to ground the results in various data sets. An empirical exploration of both the state of the art and of the needs of different stakeholders by both qualitative assessments (QUAL) and a standardized survey (QUAN) was complemented by a systematic review process (REV) as well as a series of theory of change (ToC) workshops to explore strategies for the successful implementation of PSW in the German healthcare sector.

#### 2.1.4 Integration of study parts

The various study parts were in continuous dialogue: the results of (1) the QUAL 1 phase, (2) the first ToC workshops and (3) the REV process informed the development of the QUAN survey instrument. Furthermore, the research questions and guidelines of the QUAL 2 phase derived from inconsistencies that arose during the QUAL 1 and QUAN study phases, where the qualitative data tended to attune more closely to local practices, while the QUANe study aimed at providing generalizable findings. Third, the main results of the REV, QUAN and both QUAL study parts were discussed in the last ToC workshops, and those of the QUAN, ToC and both QUAL parts were integrated in a multistep process into a guideline for PSW implementation and the main results manuscript.

### 2.2 Collaborations

#### 2.2.1 Research team

The research consortium consisted of three subteams: (1) a team at the Medical School Brandenburg (MHB) that conducted both QUAL study parts, (2) a team at the University Clinic Hamburg‐Eppendorf (UKE) that was in charge of the QUAN survey as well as the ToC and REV study parts and (3) a team based at the nonprofit organization EX‐IN Germany that served as a practice partner in various steps of the research process (see below). Furthermore, our study used (1) a collaborative research approach in combination with (2) participatory methods and strategies to engage diverse stakeholders during various stages of the research process.^49^, ^50^

#### 2.2.2 Collaborative approach

Collaborative approaches are widely used in the field of mental health.^50^, ^51^ Sometimes framed as co‐productive, they are distinct from user‐led or survivor research, which is controlled by researchers who identify as users or survivors of psychiatric services.^50^, ^52^, ^53^ However, they may, as in our case, draw on these traditions by following related principles and values, such as the distribution of agency, reciprocal partnerships and reflexivity concerning power relationships.^52^ In the context our project, researchers with and without lived experience of using mental healthcare services as well as of mental health crisis and recovery collaborated closely in all phases of the study: to develop the research proposal, the methodological framework and the research questions, to prepare and implement all assessments, to analyse and interpret the data and to disseminate our research findings. All team members were employed as research staff, drawing on different kinds of educational, professional, academic and experiential backgrounds. They occupied different positions towards the mental health system in Germany by having used/survived or been employed in mental health services, by having used or practiced PSW or having participated in related self‐advocacy or political work. Crucially, they came from diverse disciplinary fields such as mental health service research, medicine, anthropology, gender studies, nursing, cultural studies, language and literary studies, religious studies, philosophy, history, art history and psychology, making our study also a decidedly interdisciplinary one. To navigate this complexity, the labels ‘researchers with and without lived experiences (= LE)’ will be used, as this difference turned out to be central in relation to our research topic. At the same time, we are aware of the critique surrounding the concept of ‘lived experience’, both in itself^54^, ^55^ and of its application in relation to PSW.^54^

#### 2.2.3 Participatory strategies

In addition to the collaborative approach, participatory workshops were employed during various study sections and research phases. Given that a large majority of the literature on PSW implementation comes from the United States, Australia, the United Kingdom and Canada,^11^, ^12^, ^13^, ^14^, ^15^, ^16^, ^17^, ^18^, ^19^, ^56^ we found that important factors in the German healthcare context were either not addressed (such as length of training, curricula and competencies acquired, institutional or and staff preparation, PSW's agency, etc.) or formulated too vaguely to contribute to a methodological framework that would be valid for the German context. Thus, the study consortium decided to involve a diverse range of expert stakeholders related to PSW implementation in Germany (PSWs working within or outside the healthcare system, mental health staff and users, policy or political representatives, self‐help and self‐advocacy representatives) to check, refine and validate the research questions (more details below).

### 2.3 Population

#### 2.3.1 Stakeholder groups

The participants for both the QUAN and QUAL study parts were drawn from three stakeholder groups: (1) PSWs currently or previously working in the mental healthcare sector; for instance, in psychiatric hospital departments or integrated care treatment teams, (2) users of PSW from these sectors and (3) employees from other professional groups that work alongside PSWs, trained in psychiatry, psychology, social work or nursing, as well as management staff. The inclusion of these participants was intended to generate a multiperspectival body of knowledge regarding the requirements of and challenges to the implementation of PSW in the German mental healthcare sector.

#### 2.3.2 Recruitment

The recruitment took place during the study months 6–30, spanning the Years 2021–2023 and used diverse strategies, described below. It was facilitated through the largest PSW‐Training Network EX‐IN Germany and smaller PSW training organizations (EUTB, Lebensart Münster, UPSIDES, etc.), as well as through self‐help initiatives and professional networks. Additionally, information on the study was shared on PSW‐related social media platforms to facilitate the recruitment of study participants.

#### 2.3.3 Settings

Participants were recruited from all 16 federal states. To account for the diversity of services provided in different regions, special attention was paid to recruiting participants from both rural and urban areas, as well as from the North, South, East and West of Germany. This was considered necessary in the German context, given the considerable regional and institutional differences in services provided and the implementation of PSW. Details on our concrete sample strategies will be described in the following sections.

### 2.4 Assessments and methods

Due to the coronavirus disease 2019 pandemic, all qualitative and quantitative assessments were conducted online.

#### 2.4.1 Qualitative assessments (QUAL 1)

##### Overarching research questions

To initiate the research process, the overarching research questions for both the QUAL and QUAN study parts were developed in collaboration with the MHB and UKE teams. They were discussed in a participatory workshop involving 11 stakeholders (described above) and adapted accordingly. The final version of the research questions can be found in the electronic annex.

##### Development of guidelines

To develop the interview guides for the QUAL 1 assessments, the MHB team split up into smaller groups, which brainstormed around 280 potential items on the basis of their expertise as users (in the case of the researchers with LE), PSWs or mental healthcare staff, as well as their academic or professional backgrounds. The items were systematically sorted in several steps, being assessed in relation to central classificatory domains found in literature. This process was time‐consuming and required close coordination between all researchers involved. Dialogues were intense due to the diverse backgrounds and positionalities outlined above.

A total of 76 interview questions resulted, which spanned themes such as PSWs' trainings and competencies, institutional requirements before their implementation and institutional onboarding, PSWs' tasks, and roles, interprofessional collaboration, strategy and mission statements, possibilities for the PSWs' participation on an institutional level, policy development, cooptation and so on. These questions were separated into six interview guides (three for PSWs, two for other staff and one for service users, with 10 participants recruited for each guide = 30 PSW, 20 staff and 10 users) and discussed in three participatory focus groups with a total of 29 stakeholders from various backgrounds. This resulted in the modification of many questions as well as whole groups of questions.

##### Sampling

A total of 57 participants (32 PSWs, 19 staff and 6 service users) were recruited for the QUAL 1 phase via the institutions and networks described above, using a combination of snowball sampling and purposive case selection that aimed at a balanced sample from all stakeholder groups, sociodemographic backgrounds, regions and healthcare sectors. To enable a dialogical interview situation, the interviews were conducted in pairs, ideally consisting of two team members with and without LE, respectively. Interviews were recorded and anonymized during transcription, which was implemented by a corporation commissioned for this purpose.

##### Analysis

After transcription, a multistep coding process was implemented by the collaborative MHB team (see Table 1): after a coding‐as‐trial process to negotiate the scope of the coding sections and depth, each interview was coded by two team members, with and without LE, respectively. Following the coding approaches of two earlier projects,^57^, ^58^ they used a variant of thematic analysis adapted to collaborative research designs.^59^ The resulting individual codes were systematically merged in several steps: First, two groups were formed, one integrating the codes developed by researchers with LE, and the other merging those developed by researchers without LE, to enable a systematic comparison between their potentially divergent perspectives and systems of knowledge. During this process, extensive collaboration between the team members was necessary to iteratively identify, structure, approve, expand and gradually integrate the emerging themes into two overarching code systems. Then, these code systems were merged into one. This was a time‐consuming process that required extensive reflection on word choice and debates about shared meaning, aiming to avoid hierarchical processes of decision‐making. The themes of the two code systems were compared, changed, merged or adapted. Finally, a coding guide was gradually integrated, which explicated code definitions and anchor citations, thus enabling the recoding of the transcripts deductively by two team members, who remained in conversation with each other in the event of uncertainties or questions.

**Table 1:** Multistep, collaborative coding process of the QUAL 1 phase.

1	Recurrent reading of the transcripts by the full research team
2	Coding‐as‐trial in the full research team to negotiate the scope and depth of coding sections
3	Inductive coding of each transcript by two team members, with and without LE
4	Structuring of the codes into two, overarching code systems, one for the codes of the team members with and one for those of the team members without LE
5	Approval of these code systems within these groups on the basis of the inductive codes of step 3
6	Gradual merging of the two code systems by comparing and adapting their overarching themes, or structuring and integrating new ones
7	Approval of this system with the full research group on the basis of the inductive codes of step 3
8	Explication of the code definitions and anchor citations and recoding by two team members

Abbreviation: LE, lived experience.

#### 2.4.2 Systematic review (REV)

##### Collaboration

A systematic review of the existing literature on the implementation of PSW was conducted to establish an evidence‐based overview of the current state of the field. This process was mainly implemented by two UKE team members with and without LE respectively.

##### Search strategy: Academic literature

After identifying relevant keywords and databases, and to ensure the relevance of the results for the German healthcare context, the decision was taken to streamline the process by only focusing on PSW implementation in the context of European countries. Several medical databases (PubMed, Psychinfo, CINAHL, Cochrane Library, Campbell Collaboration) were searched for relevant literature published in English and German between 2010 and 2023.

##### Search strategy: Grey literature

Additionally, we searched for grey literature from that period. The latter search process enabled the inclusion of own‐voice material from PSWs, as well as materials issued by policymakers, professional societies, peer/user organizations and relevant BA and MA theses. It was a complex process including the search of various databases, professional websites, web presences of self‐advocacy initiatives and peer/user organizations and relevant library catalogues, as well as a Google Scholar search. More detailed information on this search process and its results will be published in a separate article.

##### Overall approach and application in the subsequent research process

The systematic review included peer‐reviewed articles and grey literature resources, allowing for a comprehensive synthesis of different types of evidence and relevant lines of inquiry that supported the preparation of both the ToC workshop and the QUAN and QUAL 2 assessments.

#### 2.4.3 Standardized survey (QUAN)

##### Participatory development

A standardized survey was prepared with the purpose of obtaining a diverse and comprehensive overview of the different stakeholder perspectives across Germany. The survey items were developed on basis of the QUAL 1 results, the first ToC workshops and the REV results, as well as in collaboration with the participatory board EmPeeRie Now (Empower Peers to Research^60^), a group of service users, relatives, practitioners and healthcare professionals who provide feedback on research projects in the field of mental health research, giving us recommendations on language and organization/order of items and supporting us by filling in the survey in the context of a pretest.

##### Finalization, definition of stakeholder groups and available formats

A stepwise development process reduced the items to quantifiable or free text items, the former using either Likert scales or closed questions for their evaluations. The survey was developed and published in four versions, each one addressing a different stakeholder group: (1) PSWs; (2) service users; (3) working‐level, nonpeer staff; and (4) management‐level staff. It was made available for use on lap‐/desktops or on mobile devices, as well as in a pen and paper version.

##### Recruitment strategy

In the QUAN study phase, a snowball sampling strategy was used to recruit PSWs and service users: as the largest actor in the German mental healthcare sector, all hospital departments providing services for adults were systematically approached and asked that the questionnaire be distributed to all PSW, other staff and service users. A target size of 200 people was set for each of these stakeholder groups. Further, as described above, the survey was circulated via various peer and self‐advocacy organizations, relevant community services and professional societies in Germany, as well as within team members' own social networks. The results of this recruitment strategy and its impact on the outcomes of the survey study will be detailed in the main publication of this study.

##### Analysis

Free text items were categorized using the qualitative content analysis according to Mayring and Fenzl^61^ the quantifiable data was analysed through SPSS using descriptive statistics and mean comparisons, specifically the Kruskal–Wallist test.

#### 2.4.4 Developing a ToC

##### Method

A ToC aims to support the development and implementation of an intervention by bringing together key stakeholders for a structured process of sharing perspectives and ideas to understand and examine diverse approaches and assumptions in relation to the impact of the intervention.^62^ During this process, a ToC workshop uses a reverse chronology: as starting point, it takes a previously defined desired impact (in the case of our study, this was ‘PSW has been implemented sustainably in mental health care in Germany, and is always available for service users, without barriers’), and then works backward to identify factors relevant for achieving this goal. Thereby, this method has the potential to provide a process and outcomes framework, which reflects the complex interdependencies that are entailed in an implementation, change or evaluative process. In the case of this study, a total of six ToC workshops (with between 8 and 13 participants, each) were carried out with participants from various educational, professional and experiential backgrounds. The results of these workshops will be published in a separate paper.

##### Analysis

They were visualized using a ToC map, which is a nonlinear approach revealing the interaction of different components and factors of and for the implementation of PSW, showing what changes are needed, what barriers exist and how these can be collaboratively evaluated to achieve long‐term goals.

#### 2.4.5 Qualitative assessments (QUAL 2)

##### Aims and parts

According to our research proposal, the QUAL 2 assessment aimed at further exploring questions and inconsistencies that had arisen from the earlier parts of the study. To allow for more flexibility in the choices of methodology and research questions, the MHB research team was split into several subteams to host 10 subprojects that explored a diversity of new questions, using a variety of methods, anchored in different disciplinary fields and positions: for instance one subteam conducted in‐depth research on financing models of PSW, as the previous material did not provide enough information on this topic. One researcher with LE implemented participatory workshops on the question of collective knowledge, as this dimension had hitherto remained unmentioned. Another researcher with a background in mental health nursing had recognized that the concerns of staff with LE were missing, leading to interviews and a focus group on this topic. A psychiatric researcher addressed the question of the influence of the medical model on the implementation of PSW. Another researcher with LE reanalysed coded material on the use of metaphors in the formation of PSW.

##### Sampling and realization

Depending on the various methodologies and research questions, some of these projects rerecruited participants from the first QUAL 1 phase, whereas other participants were freshly sampled using purposeful^63^ or theoretical strategies.^64^ A total of 19 focus groups, 21 expert interviews and three participatory workshops were carried out with 76 participants, among them 42 PSWs and 34 staff. These were transcribed and analysed using various analytical methods such as thematic analysis^59^ and metaphor analysis as well as MAXQDA as a coding programme.

## 3 DISCUSSION

As our study largely builds on transdisciplinary, multiperspectivist teamwork, the knowledge produced emerged across fluid disciplinary and experiential boundaries and divergent, often ambivalent positionalities, also towards PSW. Most importantly, the various participatory formats used throughout the project helped to ensure that concepts and framings captured the complex and frequently contrasting practices, philosophies and policy debates concerning local PSW implementation. Thus, the research itself was a social process that actively shaped the phenomenon we studied—an understanding that has also been proposed in relation to research on PSW more recently.^37^, ^38^ This process involved frequent methodological debates in our research team concerning the nature of the research topic and appropriate ways to frame it. Through these debates, the focus of our analyses was shaped step‐by‐step, foregrounding certain aspects of the research object, while devaluing others, which were then subject to revision and reflection once again.^65^

The following discussion will highlight some of these methodological debates: in the first part, the formation of the research topic will be explored in relation to its political and funding conditions. Subsequently, it will be investigated in how far the methodological decisions taken during the project have iteratively shaped the object of our study. And in the third part, the impact of the collaborative and participatory design will be critically discussed. Thereby, the discussion is grounded in the assumptions that: (1) the production of knowledge is always contingent to a specific social or institutional context^66^; and (2) that a research process is to be perceived as a series of social decision‐making events,^67^ also emerging from the methodological framework chosen, and operationalizing the epistemic object at hand in only one of many possible ways.

### 3.1 Formation of an epistemic object…

Proposing a research project on German‐wide PSW activities had been of interest to many of the co‐authors of this paper for years. Various ideas and research approaches had been exchanged but were dismissed either due to problems of feasibility or lack of funding. Thus, the research topic of our study was not self‐evident, but rather highly contingent on (1) the political situation given and (2) the conditions of the funding agency involved.

The proposal tied to the historical development of the already mentioned PPP‐RL law, which made it necessary to develop PSW‐specific staffing guidelines. Our focus on implementation, therefore, was a strategic one in response to this political situation, but not without epistemic consequences: it restricted our research to the analysis of PSW *within* the mental healthcare system, leaving almost no space for exploring alternatives, not only from the psychosocial sector (in which most of the PSWs work) but also within the context of self‐help and self‐advocacy approaches to peer support that usually are marginalized in the German healthcare context.^68^, ^69^ This is equally true for the alternative, autonomous support practices of the c/s/x‐movement that usually remain unmentioned in studies on PSW internationally, despite often being proposed as safeguards for its implementation in clinical settings^70^—a paradox that reflects the silencing of this marginalized expertise.

To mention this limitation to our study is especially important as international research highlights how PSW in clinical settings has come to dominate the practices and understandings of peer support more generally.^71^, ^72^, ^73^ An unintended consequence of this development has been the withdrawal from funding for other types and approaches of peer support, for instance from self‐help initiatives.^71^, ^72^, ^73^ There is a dark irony at play here, as peer support was originally developed autonomously within the c/s/x‐communities to resist the psychiatrization of mental distress and the paternalism ingrained in the mental healthcare system.^20^ Now, deriving benchmarks for the implementation of PSW from the very system that these forms of peer support originally objected to, carries the risk that insights from studies such as ours may be used to define and regulate other approaches of peer support in nonclinical settings too.

These and other questions became ever more strenuous due to the various standpoints towards PSW in clinical settings within the MHB research teams. At the same time, these differences enabled the emergence of complex arguments concerning questions such as: is our methodological framework suitable for the exploration of the distinct principles and values of peer support? Does it do justice to its emancipatory roots, or does our project contribute to the silencing of less visible, marginalized initiatives of peer support? In our search for answers to such questions, we repeatedly worked to subvert the authority of our research proposal: the division of the MHB team into subteams in the QUAL 2 phase can be understood as one attempt in this direction, the discussion of these dilemmas in the context of this manuscript is another.

### 3.2 … and its operationalization

During the implementation of our 3‐year project, our methodological framework was continuously adapted, which, in turn, modified the object of our study. Each methodological decision can be understood as a step towards its further operationalization, both enacting and mediating its epistemic and political impact.

Due to the contingencies described above, the *research questions* of the QUAL 1 phase had to focus on the improvement, institutionalization or professionalization of PSW in the context of the mental healthcare system, instead of questioning the fundamental logic behind such an integration. Despite our desire for more critical inquiry in these directions—and notwithstanding critical voices among the participants in our assessments or workshops—this overarching structure left little space for extended emancipatory scrutiny. It is difficult to include critical standpoints via a handful of items added to an interview guide or questionnaire.^74^ Further, mental health service research largely focuses on optimizing or improving health care, leaving considerably fewer possibilities for deconstructing or fundamentally reorganizing it.^38^

Regarding our *sampling strategy*, we had difficulties sampling participants who had either dropped out or decided against working in this system in the first place. Further, a large part of recruitment was mediated by PSWs or staff that were highly committed to the integration of PSW in the healthcare system, serving as gatekeepers that further shaped the composition of our study population. Using snowball sampling in the QUAN study part to recruit service users and PSWs contributed to this self‐selection as well. Truly critical perspectives or ‘angry voices’ that conflict with mental healthcare regimes of treatment and diagnoses^75^ were certainly present, but relatively rare in the context of our study.

Furthermore, the *selection of methods* shaped the object of our study: for instance, the use of ethnographic methods, such as participant observation would have certainly yielded different results. What is articulated in relation to PSW during an interview may deviate considerably from what happens in practice, given the highly normative discourse that circulates around PSW, at least in Germany.^8^ Thus, the lack of focus on the complex and contradictory entanglements of practical knowledge and daily interaction, brought forward by ethnographic investigations,^54^ remains a central limitation of our study.

Finally, the *analysis of data* was also vulnerable to various contingencies: the use of thematic analysis in the QUAL 1 phase allowed less for conceptual, in‐depth or critical inquiries than, for instance, a grounded theory analysis would have done. Yet, this method seemed necessary considering the sheer number of interviews to be analysed— a number that may not be unusual for medical or health service research but is rather high in the field of emancipatory research.^52^

### 3.3 Impact of collaboration and participation

The decision to employ a collaborative and participatory research framework shaped the emergent understanding of our object of study as well, something which has been similarly described in other projects.^74^ The staffing of all subteams by researchers of various disciplinary, professional and experiential backgrounds was sometimes a challenge, but mainly a strength. It enabled the generation of divergent and novel interrogations of the research problem at hand, bringing to the fore questions and analytical strands that would not have been thought of in a noncollaborative projects. Further, the inclusion of divergent perspectives during the participatory and ToC workshops was equally broadening our investigative scope and analytical spectrum.

On the other hand, given the project's limited resources, the interdisciplinarity and multiperspectivity of our methodological framework made it necessary to employ all team members in part‐time positions. This composition resulted in the considerable dispersion of the team structure, sometimes making everyday tasks like scheduling disproportionately difficult and maintaining the equity and sustainability of workloads an ongoing challenge. These concerns were more immediately relevant to the MHB team, which consisted of more researchers than the relatively small UKE team. Yet, it left fewer options for close collaboration between the entire team too than conceptualized in the original research proposal.

Team supervision is vital for this kind of research.^74^ In our study, it offered a space for reflection on the team's dynamics and their impact on our understanding of the object of study. Concerns regarding hierarchies (both formal and informal ones), power relations, debates about language use, politics, burnout and ethical issues regularly featured in these supervision sessions, and influenced the ways in which we came to think about PSW implementation. Thus, our team's diverse personal, professional and disciplinary backgrounds provided a reflexive infusion of different positionalities into the production of knowledge. Crucially, this complex journey, while leading to more comprehensive and compelling results, required substantial emotional processing that was not accounted for in our research proposal.

## 4 CONCLUSION

Taking stock of the complexity and challenges of our methodological framework, three conclusions can be drawn. First, the wider political or funding context impacts the research questions chosen, as in our case, the focus on PSW implementation. Second, this manuscript demonstrates that the choice of methods, the ways in which study subjects are selected and the strategies used to analyse data may iteratively shape the research object to be studied, that is, the ways in which a research team thinks of or operationalizes the topic of concern. Third, the use of a collaborative research approach or participatory strategies may equally contribute to the formation of an epistemic object by steering the analytical spectrum in directions that would have been inconceivable in noncollaborative projects.

In relation to the third conclusion and the topic of implementation of PSW, we argue that a collaborative and participatory research framework is strongly recommended, as insights on this topic often emerge from lived experiences. Such an approach may generate divergent and novel analytical strands that could be overlooked in noncollaborative/participatory projects. As PSW originally emerged from emancipatory initiatives of self‐help and self‐advocacy, which were and often still are critical of the mental healthcare system, a medical or mental health service research framework is usually not suited to capture these important critiques.

## AUTHOR CONTRIBUTIONS

**Sebastian von Peter:** Conceptualization; investigation; writing—original draft; funding acquisition; methodology; formal analysis; supervision; resources; project administration. **Ute Maria Kraemer:** Conceptualization; writing—original draft; formal analysis; investigation. **Lauren Cubellis:** Investigation; writing—original draft; methodology; writing—review and editing; conceptualization. **Georgia Fehler:** investigation; writing—review and editing; formal analysis. **Guillermo Ruiz‐Pérez:** investigation; writing—review and editing; formal analysis. **Daniela Schmidt:** Investigation; writing—review and editing; formal analysis. **Jenny Ziegenhagen:** Investigation; writing—review and editing; formal analysis; funding acquisition. **Madeleine Kuesel:** Investigation; writing—review and editing; formal analysis. **Susanne Ackers:** Formal analysis; data curation; writing—review and editing; validation. **Candelaria Mahlke:** Writing—original draft; writing—review and editing; methodology; formal analysis; supervision; project administration; conceptualization; funding acquisition. **Lena Nugent:** Formal analysis; writing—review and editing; writing—original draft; investigation; conceptualization. **Imke Heuer:** Conceptualization; writing—original draft; writing—review and editing; formal analysis.

## ETHICS STATEMENT

Ethics were approved by the ethical committee of the Medical School Brandenburg (E‐01‐20200826).

## Supporting information

Supporting information.

## Data Availability

Data sharing is not applicable to this article as no new data were created or analysed in this study.
