# Supplementary material for: Implementing peer support work in mental health care in Germany: The methodological framework of the collaborative, participatory, mixed‐methods study (ImpPeer‐Psy5)
Source: Health Expect. 2023 Dec 19;27(1):e13938. doi: 10.1111/hex.13938 (PMC10729536; doi:10.1111/hex.13938)
Supplement: Supplementary file 1 — Supporting information. [file HEX-27-e13938-s001.docx]

**Overarching research questions**

| **Thematic Fields/**  **Moduls Proposal** | **Individual Requirements (A)** | **Expectations of the PSWs (B)** | **Struktural Requirements (C)** | **Tasks und Roles (D)** | **Perceived Changes (E)** | **Peserving the specifities of PSW (F)** |
| --- | --- | --- | --- | --- | --- | --- |
| **Current Practices:**  **How is PSW currently implemented in Germany?** | What competences (experience, training, skills) do the PSWs bring to their work? | What expectations do different stakeholders have of PSW? | What provisions have been made for the implementation and further development of PSW? | Which tasks are carried out by the PSWs, which roles are assumed? | How does the implementation of PSWs affect their institution and users? | What is being done in the institutions to preserve and develop the specific values, principles, and modes of operation of the PSWs? |
| **Nees and Requirements: What are the needs of PSWs and other stakeholders in terms of implementation?** | What competences (experience, training, skills) should the PSWs have? | What expectations should the stakeholders have PSW? | What requirements would be needed for the implementation and further development of PSW? | Which tasks and roles should be performed by PSWs and which roles should be taken? | Which changes should PSWs effect in the institutions? | What is needed in the facilities so that the specifities of the PSW is preserved and developed? |
| **Implementation Strategies: Which models of PB are suitable or conducive for supply in Germany?** | What competences (experience, training, skills) of the PSWs are necessary or beneficial for their work in the mental health care sector? | What are the stakeholders' expectations of PSW in the mental health care settings? | Which requirements are necessary / conducive for the implementation and further development of PSW in the mental health care sector? | Which roles and tasks are suitable for the implementation of PSW in the mental health care sector? | What changes can PSW effectuate in mental health care institutions? | What is necessary/promoting to preserve and further develop specific values, principles and modes of operation of PSW? |

Legend: PSW = Peer Support Work/ PSWs= Peer Support Workers
